# On the Nature of Extra-Framework Aluminum Species and Improved Catalytic Properties in Steamed Zeolites

**DOI:** 10.3390/molecules27072352

**Published:** 2022-04-06

**Authors:** Konstantin Khivantsev, Nicholas R. Jaegers, Libor Kovarik, Miroslaw A. Derewinski, Ja-Hun Kwak, Janos Szanyi

**Affiliations:** 1 Institute for Integrated Catalysis, Pacific Northwest National Laboratory, Richland, WA 99352, USA; ncderewi@cyf-kr.edu.pl; 2 Jerzy Haber Institute of Catalysis and Surface Chemistry, Polish Academy of Sciences, 30-239 Krakow, Poland; 3 Department of Chemical Engineering, Ulsan National Institute of Science and Technology (UNIST), Ulsan 44919, Korea

**Keywords:** zeolite, extra-framework aluminum species in zeolite EFAL, cracking and dehydrogenation in zeolites, steamed zeolites, infra-red spectroscopy

## Abstract

Steamed zeolites exhibit improved catalytic properties for hydrocarbon activation (alkane cracking and dehydrogenation). The nature of this practically important phenomenon has remained a mystery for the last six decades and was suggested to be related to the increased strength of zeolitic Bronsted acid sites after dealumination. We now utilize state-of-the-art infrared spectroscopy measurements and prove that during steaming, aluminum oxide clusters evolve (due to hydrolysis of Al out of framework positions with the following clustering) in the zeolitic micropores with properties very similar to (nano) facets of hydroxylated transition alumina surfaces. The Bronsted acidity of the zeolite does not increase and the total number of Bronsted acid sites decreases during steaming. O_5_Al(VI)-OH surface sites of alumina clusters dehydroxylate at elevated temperatures to form penta-coordinate Al_1_O_5_ sites that are capable of initiating alkane cracking by breaking the first C-H bond very effectively with much lower barriers (at lower temperatures) than for protolytic C-H bond activation, with the following reaction steps catalyzed by nearby zeolitic Bronsted acid sites. This explains the underlying mechanism behind the improved alkane cracking and alkane dehydrogenation activity of steamed zeolites: heterolytic C-H bond breaking occurs on Al-O sites of aluminum oxide clusters confined in zeolitic pores. Our findings explain the origin of enhanced activity of steamed zeolites at the molecular level and provide the missing understanding of the nature of extra-framework Al species formed in steamed/dealuminated zeolites.

## 1. Introduction

Zeolites are some of the most important industrial materials employed in petroleum refining and cracking [1,2,3,4,5,6,7,8,9]. These crystalline and microporous solids are composed of tetrahedral SiO_4_ and Si-OH-Al units with ordered arrays of micropores/microcavities. Alkane transformations into more valuable chemicals take place in zeolitic pores at high temperatures 400–650 °C [1,2,3,4,5,6,7,8,9]. Similar to transition aluminas, zeolites are industrially produced with a yield of millions of tons every year [1,2,3,4,5,6,7,8,9]. For hydrocarbon cracking, it is well known that mildly steaming zeolites at elevated temperatures significantly improves their cracking activity [1,2,3,4,5,6,7,8,9,10,11]. This phenomenon, although studied extensively, has remained poorly understood for the last six decades. It is generally known that zeolite steaming generates extra-framework Al (EFAL) species, and it is often speculated that the presence of EFAL species increases the Bronsted acidity of zeolites, which contributes to increased protolytic cracking activity [11,12]. Comprehensive reviews of the literature, however, conclude that no convincing evidence exists of increased Bronsted acidity after hydrothermal treatment [13].

Previously, through a combined microscopy and spectroscopy approach, an expanded understanding of the structural and chemical properties of γ-Al_2_O_3_ surfaces has led to revised assignments for some IR features of surface OH groups [14]. Specifically, it was shown that the OH band at ~3770–3780 cm^−1^ belongs to amphoteric O_5_Al(VI)-OH sites associated with nano-segments of restructured (110) surfaces because (110) surfaces of gamma alumina restructure into (100) and (111) nano-segments; although the macroscopically defined (110) surface exists, its atomic-level view shows that it is completely broken into (100) and (111) nano-segments, due to the higher thermodynamic stability of (100) and (111) facets. This holds true for nanosized commercial high surface area (up 200 m^2^/g) SBA-200 gamma alumina as well as larger and well-defined rhombus-platelet gamma alumina crystals; Figures S1 and S2.

## 2. Results and Discussion

**Figure 1:**
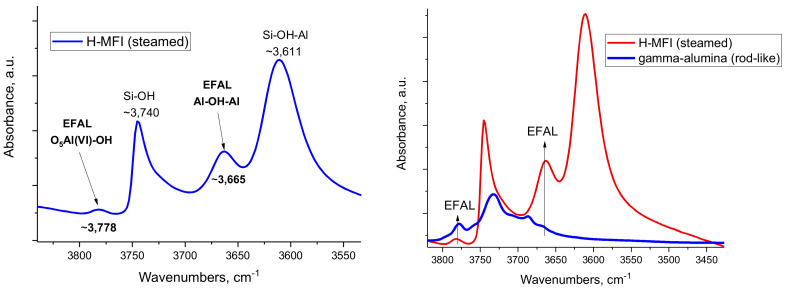
FTIR spectra of MFI and γ-Al_2_O_3_ catalyst materials. (**Left**) FTIR spectrum of the OH region of steamed H-MFI sample. (**Right**) FTIR spectrum comparison of the OH regions of steamed H-MFI and rod-like gamma alumina sample.

As previously shown [14], the OH IR bands between 3720–3740 cm^−1^ belong to tetrahedral O_3_Al-OH sites. Bands below 3690 cm^−1^ belong to weakly acidic doubly and triply bridging OH groups [Al--OH--Al] of gamma alumina [14]. It was also shown that alcohol dehydration catalytic activity stems from the presence of O_5_Al(VI)-OH sites [14]. Upon thermal dehydroxylation, O_5_Al-OH sites transform into coordinatively unsaturated O_5_Al sites that can bind both N_2_ and CO. This new understanding allows for elaboration on the nature of extra-framework Al species formed in zeolites by drawing parallels between the EFAL species in the zeolite channels and transitional γ-Al_2_O_3_. More specifically, after steaming all studied zeolites (H-SSZ-13, H-ZSM-5, and H-BEA), they produced the signatures of extra-framework species, i.e., the bands in the -OH stretching region at ~3770–3780 cm^−1^ and ~3660–3640 cm^−1^ (Figure S3). The 3770–3780 cm^−1^ band is often ignored because it is less intense and is present as a tail on the O-H vibration of silanol groups [15]. The similarities between the signatures of OH groups on the surface of gamma alumina and steamed zeolites suggests that the EFAL species formed in zeolites possess OH groups with very similar properties to those of transition aluminas. (Figure 1).

**Figure 2:**
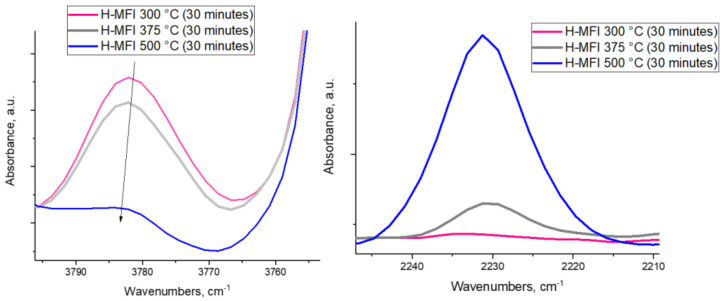
FTIR spectra of steamed H-MFI sample (all spectra were collected on the same pellet) (**Left**) OH region showing the diminishment of the ~3780 cm^−1^ band with temperature (spectra were recorded at room temperature). (**Right**) FTIR final spectra collected after introducing a total of 1 Torr of CO (total pressure, room temperature) on the steamed H-MFI sample after each thermal treatment and decreasing to room temperature. The development of Al-CO spectra sequentially during in-situ N_2_ and CO adsorption is shown in Figure 4.

**Figure 3:**
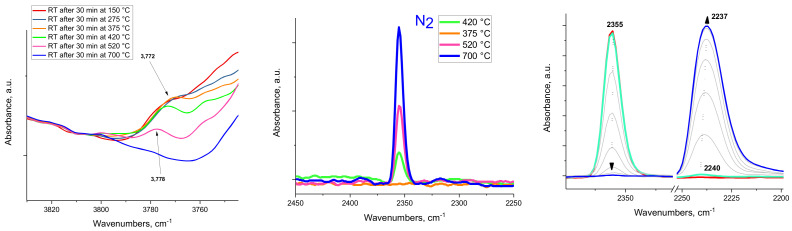
IR spectra for a rhombus-platelet gamma alumina sample. (**Left**) FTIR of the OH region showing a diminishment of the ~3780 cm^−1^ band with an increase in temperature (spectra were recorded at room temperature after each treatment). (**Middle**) FTIR spectra collected after introducing a total of 5 Torr of nitrogen (total pressure; room temperature) on the alumina sample after each thermal treatment. The evolution of the N-N stretching band is due to N_2_ adsorption on Al_1_O_5_ dehydroxylated sites which only occurs when the specific OH band decreases from dehydroxylation taking place. (**Right**) Sequential CO adsorption on the sample containing a O_5_Al-N_2_ complex: this establishes that CO displaces N_2_ as a coordination site in the O_5_Al-CO complex. This complex has a CO stretch around ~2240–2236 cm^−1^, very similar to the surface Al_1_O_5_ sites of dehydroxylated Al_2_O_3_ clusters in zeolites. The spectra of dehydroxylation of SBA-200 and rod-like alumina samples are shown in Figure S4.

**Figure 4:**
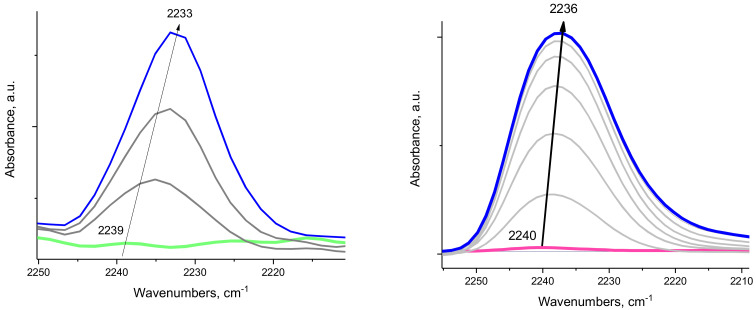
(**Left**) FTIR during sequential CO adsorption (~1 Torr) on a steamed H-MFI sample after dehydroxylation at 500 °C. (**Right**) FTIR during sequential CO adsorption (~1 Torr) on a rod-like gamma alumina sample after dehydroxylation at 500 °C.

Next, we demonstrate that EFAL species in zeolites are aluminum oxide clusters (formed upon steaming) and that they possess similar surface properties as typical nano-segmented aluminum-oxide surfaces. As the steamed H-MFI sample was heated above ~400 °C, the intensity of the ~3780 cm^−1^ OH band decreased (left panel of Figure 2). Simultaneously, coordinatively unsaturated penta-Al sites were produced and their presence was substantiated by the development of an IR feature at ~2239–2233 cm^−1^ upon exposure to CO after decreasing the temperature to room temperature (right panel of Figure 2). CO adsorption on the steamed, but not thermally dehydroxylated, H-ZSM-5 sample (where the 3780 cm^−1^ band was present) did not lead to significant CO or N_2_ adsorption (Figure 2). An identical phenomenon was observed for various alumina samples (Figure 3 and Figure S4) treated thermally. As the intensity of the 3778 cm^−1^ band decreased (note that the location of this band is very close both for alumina (100), (111) segments and the steamed zeolite [14]), the ~2233–2236 cm^−1^ band developed after CO adsorption (Figure 3). Moreover, its intensity varied in a similar fashion for both the dehydroxylated alumina surfaces and the dehydroxylated, steamed zeolite (Figure 4). It should be noted that N_2_ adsorption on dehydroxylated Al_2_O_3_ clusters in zeolites produced the N-N stretch at ~2353 cm^−1^ at room temperature, analogous to N_2_ adsorption on the transition alumina surfaces with ~2256 cm^−1^ N-N band.

As with gamma alumina, the intensity of the band of the Al-CO complex at 2239 cm^−1^ of the steamed H-MFI zeolite increases with the extent of dehydroxylation (Figure 4). This suggests that there is equivalency between hydroxylated EFAL species and hydroxylated alumina segments that dehydroxylate with the formation of coordinatively unsaturated Al_1_O_5_ sites. This provides direct evidence that the hydroxylated Al_2_O_3_ clusters that are formed in zeolite during steam treatment have similar Al-OH sites to the ones seen on transition (gamma) alumina surfaces. Furthermore, they also dehydroxylate similarly, producing coordinatively unsaturated Al_1_O_5_ sites with similar coordination/chemisorption properties (Figure 4).

The alumina clusters thus formed during the steam treatment of the zeolite are limited in size by the dimensions of the zeolite channels/cavities (i.e., < 1 nm), and thus primarily consist of surface sites. This explains the presence of a high fraction of penta-coordinated Al^3+^ sites observed in steamed zeolites by ^27^Al MAS-NMR (Figure S5). We have previously shown that penta-coordinated Al^3+^ sites are located on nano-segments of gamma alumina (Figures S1 and S2) TEM images. Such segments can be as small as a few atoms (Figures S1 and S2). A majority of the atoms in regular alumina samples are contained within the bulk oxide structure as tetra- and octahedral Al sites. Even alumina samples with relatively high surface areas contain below a few percent of penta-coordinate Al^3+^ sites (Figure S6), reflected by the low contribution of the surface to the total number of Al sites. Remarkably, Al_2_O_3_ clusters in zeolites consist mostly of surface sites, as evident by the high abundance of penta-sites, due to their nano-sized nature, resulting in an increased number of Al_1_O_5_ (after dehydroxylation) and O_5_Al(VI)-OH sites (before dehydroxylation). Accordingly, in steamed H-MFI, the ^27^Al NMR signals of tetra- and octahedral Al^3+^ sites broaden in comparison to those in fresh H-MFI, consistent with alumina cluster formation and new signals developing which partially overlap with existing Al resonances. This is fully consistent with previous Al ssNMR findings for thin alumina films [16]. It is often seen in steamed zeolites that the broadening of a tetrahedral Al signal, the formation of new octa-sites, and the appearance of abundant penta-Al sites occur. The new tetra-, octa- and penta-sites belong to small alumina clusters (the tetra-Al band overlaps partially with the sharp band of framework Al and because of this, it looks visually broadened). Because penta-Al sites are present only on the surface, their high relative abundance compared to bulk alumina corroborates the small size of the alumina clusters.

As moderate steaming removes some framework Al sites and produces extra-framework alumina clusters, the propane cracking activity increases dramatically (~6 times higher rate and TOF) (Table 1), suggesting that the presence of extra-framework alumina clusters is critical for improving the cracking activity. Concomitantly, alkane dehydrogenation activity increases as well (Table 1). Please note that propane conversions are below 2% and that monomolecular cracking takes place.

**Figure 5:**
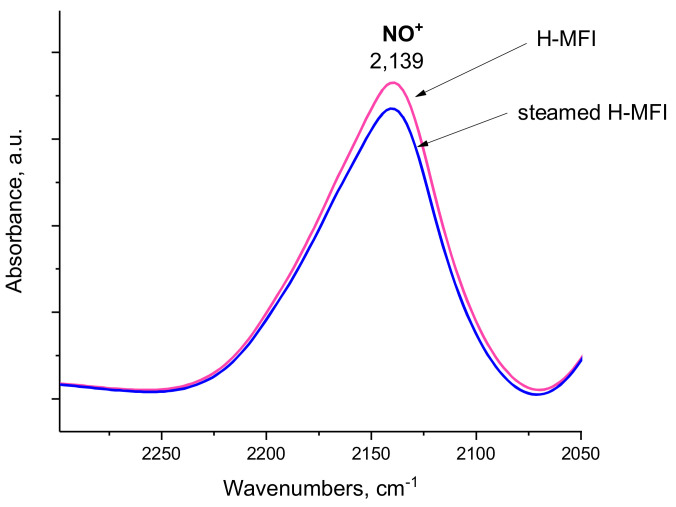
FTIR spectra in the N-O stretching region: a comparison of parent H-MFI (containing no EFAL) and steamed H-MFI (containing EFAL). NO^+^ was produced by NO_2_ adsorption (0.5 Torr in total). Because NO^+^ occupies H sites, it is a direct measure of changes in Bronsted acid quantity and acidity. The total number of Bronsted acid sites decreased after steaming, evidenced by a diminished peak area and no evidence of increased Bronsted acidity is shown (no shift in the NO^+^ stretch).

We have also monitored the change in the number of Bronsted acid sites upon steaming of the H-MFI zeolite by replacing H^+^ ions with NO^+^ ions and monitoring both the position and intensity the N-O stretching vibration in the IR spectrum [17,18,19,20]. Changes in the intensity of this IR feature can be used to monitor the variation in the number of Bronsted acid sites as a result of steaming, while the change in the position of the IR band would provide evidence for the change in acidity of the remaining acid site. The IR spectra in Figure 5 clearly show that the number of Bronsted acid sites decreased in the H-MFI sample upon steaming, while there was no change in the Bronsted acidity of the remaining OH groups. Therefore, despite the decrease in the Bronsted acid sites and no change in their acidity, the alkane C-H activation activity increased dramatically due to presence of Al_2_O_3_ clusters.

**Figure 6:**
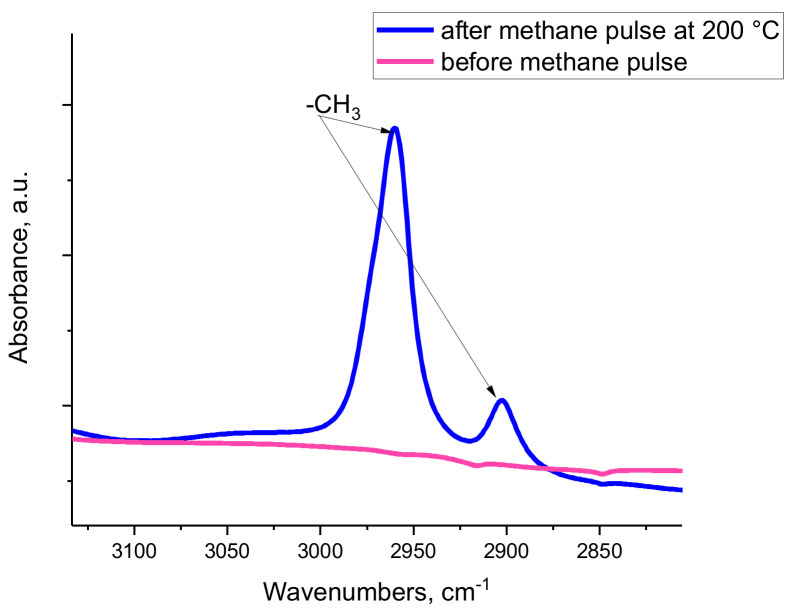
FTIR spectra showing the C-H stretching region of dehydroxylated gamma alumina before and after methane exposure (~0.5 Torr total pressure of methane) at 200 °C. The bands that developed correspond to two IR active vibrations of a methyl group.

As such, the preponderance of data suggests that Al_2_O_3_ clusters formed during steam treatment are responsible for the increased cracking activity of the steamed H-MFI zeolite, but it is not yet shown how micro-cluster domains of alumina promote this catalytic transformation. It is now shown by us spectroscopically (Figure 6), that dehydroxylated O_5_Al(VI)-OH sites (i.e., penta-coordinate Al^3+^ ions) are capable of heterolytically breaking the C-H bond of methane, the hydrocarbon with the most inert bonds and the hardest to activate, at temperatures as low as 200 °C.

Note that when the O_5_Al(VI)-OH sites were not dehydroxylated by thermal pre-treatment at high temperature (500 °C), practically no C-H bond activation was observed (Figure S7). Heterolytic C-H bond activation to form a methyl fragment apparently only occurred at temperatures where dehydroxylation, i.e., formation of Al_1_O_5_ sites, is possible (Figure 6):

Al_1_O_5_ + CH_4_ → CH_3_-AlO_4_--OH

where --OH denotes a bridging OH group between two Al atoms (the same way to denote –OH is used throughout the rest of the text). When generalized for other alkanes:

Al_1_O_5_ + C_n_H_2n+2_ → H_2n+1_C_n_-AlO_4_--OH

Specifically, for propane:

C_3_H_8_ + O_4_Al-O-Al → CH_3_-CH_2_-CH_2_-AlO^4^--OH--Al.

Please note that in that scheme Al_1_O_5_ is denoted as O_4_Al-O-Al to better show the presence of Al-O-Al bonds. The initial propane activation is followed by a beta-hydride elimination that produces propylene (C_3_H_6_) and Al hydride species:

CH_3_-CH_2_-CH_2_-AlO_4_--OH--Al → C_3_H_6_ + O_4_Al(H)--OH--Al

Hydridic hydrogen then recombines with the acidic hydrogen of the (bridging) OH group to reform the Al_1_O_5_ site and molecular hydrogen:

O_4_-Al(H)--OH--Al → O_4_Al-O-Al + H_2_

Subsequent reactions (leading to cracking products) can then occur on Bronsted acid sites, located in the vicinity of alumina clusters. Si-O(Alkyl)-Al species form from reaction of olefin with Bronsted acid sites:

C_3_H_6_ + Si-OH-Al → Si-O(C_3_H_7_)-Al

This step is critical to form the alkoxy intermediate required for cracking. Once alkoxy species are formed, they can irreversibly crack on Bronsted acid sites. Since propane dehydrogenation reaction is reversible (C_3_H_8_ ← → C_3_H_6_ + H_2_), the reaction is limited by thermodynamic equilibrium. However, because of the proximity of Al_2_O_3_ clusters to zeolite acid sites, the propene can immediately participate in further irreversible reactions on Bronsted acid sites leading to cracking products. The continuous consumption of propylene shifts the equilibrium of the reversible dehydrogenation reaction to the right, ultimately increasing propane conversion:

C_3_H_8_ ← → H_2_ + C_3_H_6_ + Zeolite-O-H → Zeolite-O-C_3_H_7_ + H_2_ → C_1_ + C_2_ cracking products

**Figure 7:**
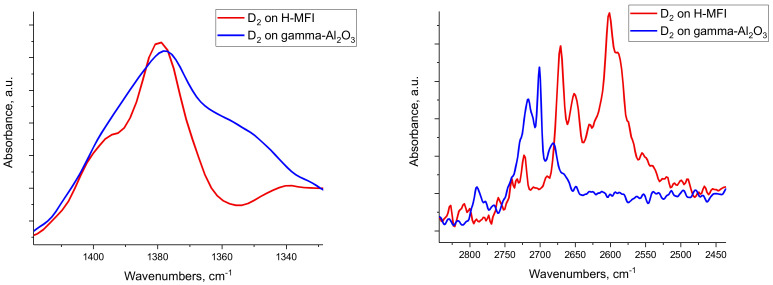
FTIR spectra in the (**left**) Al-D and (**right**) O-D stretching regions after D_2_ treatment of steamed H-MFI (denoted in the graph as H-MFI and gamma alumina). Both samples were pre-treated at 500 °C prior to reaction with D_2_.

Indeed, even gamma alumina can convert propane at 500 °C to propylene and hydrogen (Table 1). Note that it also can perform the reverse reaction, propylene hydrogenation to propane. The dehydrogenation selectivity of gamma alumina is ~90%, with the residual ~10% showing cracking selectivity to CH_4_ and C_2_H_4_ in a nearly 1:1 ratio with minor ethane amounts due to some back-hydrogenation of ethylene to ethane. The presence of Al_1_O_5_ stimulates this activity on both gamma alumina and zeolites. It follows that the catalytic cracking/dehydrogenation reactivity of zeolites is, therefore, due to the presence of Al_2_O_3_ clusters with coordinatively unsaturated Al_1_O_5_ sites proximal to Bronsted acid centers in the zeolite cavities. Furthermore, this mechanism invokes the formation of aluminum hydride species on Al_2_O_3_ clusters inside the zeolite channels. These Al-H species are proposed to be the essential intermediates in olefin hydrogenation and alkane activation reactions. Indeed, treating gamma alumina with H_2_ (Figure S8) or D_2_ (Figure 7) produces Al-H and Al-D stretches in the IR spectra, respectively, with H/D isotopic shift fully consistent with hydride formation. Note that hydrides can form on different Al-O sites, but not necessarily on an Al_1_O_5_ site (C-H breaking, however, seems to occur only on dehydroxylated coordinatively unsaturated Al-O sites of aluminum oxide clusters):

Al-O-Al + H_2_ → H-Al--OH--Al

In steamed zeolites, remarkably, D_2_ treatment leads to the formation of hydrides with essentially the same Al-D stretching frequencies as the ones observed on gamma alumina, consistent with the above description. This is the first identification of hydrides on EFAL species in zeolites.

We also note that the presence of water (i.e., OH groups) on extra-framework Al_1_O_5_ sites is detrimental for cracking rates, as the activity decreased significantly at temperatures when dehydroxylation did not take place (390 °C, for instance). This further highlights the critical role of dehydroxylated Al_1_O_5_ sites located on alumina clusters formed upon steaming and explains why at lower temperatures, the presence of O_5_Al(VI)-OH sites with an -OH group (dissociated water) is detrimental for alkane transformation activity since those sites are covered by -OH groups and, therefore, cannot facilitate C-H bond breaking.

With the insight gained from these results, we can explain how steamed zeolites can effectively catalyze H_2_/D_2_ exchange as well as other hydrocarbon/D_2_ exchange reactions to form deuterated hydrocarbons at elevated temperatures [21,22]. Previous studies suggested that a zeolitic Bronsted-acid catalyzed the process. In contrast, these findings suggest that this explanation may need to be revised, as zeolites with no or small amounts of EFAL have lower activity for H/D exchange in the benzene + D_2_ reaction. When EFAL species (shown here as Al_2_O_3_ clusters) are present (i.e., in steamed zeolites) this process occurs. This process takes place by: (1) Hydrocarbons dissociating on coordinatively unsaturated Al-O sites (Figure 6):

C_6_H_6_ + Al-O-Al → C_6_H_5_-Al--OH--Al

(2) D_2_ dissociating on various Al-O sites on the alumina surface (Figure 7):

Al-O-Al + D_2_ → D-Al--OD--Al

(3) Bridging protons, being weakly acidic [14], migrating between different acidic hydroxyls. Minor amounts of moisture can greatly facilitate this, which explains reported rate enhancements for H/D exchange of benzene on steamed zeolites in the presence of moisture [10]. (4) The H in an OH group then can exchange for a D proximal to Al-C_6_H_5_ site via the aforementioned proton-hopping mechanism. Finally, (5) the deuterated benzene desorbs:

C_6_H_5_-Al--OD--Al → C_6_H_5_D + Al-O-Al

This same process can occur on gamma alumina surfaces as well (see Figure S9, confirming HD formation from (H_2_ + D_2_) reaction on gamma alumina).

In summary, controlled steaming increases the cracking activity of zeolites due to the formation of small alumina clusters enriched with Al(V)_1_O_5_ surface sites in the micropores. These sites facilitate alkane C-H bond breaking events in zeolites. Simultaneously, the density of Bronsted acid Al-OH-Si sites decreases as a result of dealumination by the steam treatment. This slight decrease is also known to be beneficial because coke formation is favored by the close proximity of many Bronsted acid sites. Provided that not too much Al is removed from the framework, and that a sufficient population of Al_2_O_3_ clusters is formed, the presence of Al_1_O_5_ on their surface leads to easier C-H bond breaking events for cracking and dehydrogenation reactions. 

These findings may further suggest that the La-promotion of cracking activity in zeolites may also be related to the formation of La_2_O_3_ clusters in the zeolitic micropores. Once dehydroxylated, these clusters possess coordinatively unsaturated La-O sites, facilitating C-H bond breaking events.

Our study suggests a lack of NMR-invisible tri-coordinate Al species in mildly steamed zeolites (or on alumina surface) in measurable amounts. We have already shown in previous studies that IR spectroscopic signatures were previously incorrectly attributed to NMR-invisible tri-coordinate Al sites on the surface of transition aluminas. In fact, those sites are penta-coordinate Al^3+^ ions [14]. Additionally, the formation of tri-coordinate Al sites was previously suggested for some steamed zeolites [23] on the basis of Al K-edge EXAFS modeling. As corroborating evidence for the formation of these tri-coordinated sites, the IR band of adsorbed CO around ~2230 cm^−1^ was suggested. In our prior study on γ-Al_2_O_3_, however, we showed convincingly that this band, in fact, belongs to CO molecules associated with penta-coordinate, and not 3-coordinate, Al^3+^ sites [14]. Some recent studies further explained the enhanced alcohol dehydration activity in steamed zeolites by the presence of extra-framework tri-coordinate Al sites. Our data, however, suggests that the enhanced activity in alcohol dehydration for steamed zeolites can be explained by the presence of hydroxylated alumina clusters with abundant surface O_5_Al(VI)-OH sites. These sites have been identified as the active centers for alcohol dehydration reactions on γ-Al_2_O_3_. Furthermore, studies that proposed the presence of tri-coordinate Al sites in steamed zeolites, as probed by NMR spectroscopy with P(CH_3_)_3_ and O=P(CH_3_)_3_ probe molecules, may need to be reassessed [24]. The NMR signature ascribed to tri-coordinate Al sites in those studies likely belong to strongly Lewis acidic penta-coordinate Al^3+^ sites [14,25].

## 3. Materials and Methods

MFI in the ammonium form with a Si/Al ratio ~15 was supplied by Zeolyst. First, this sample was treated in the dry air flow at 550 °C for 5 h to remove ammonia. The sample was subsequently subjected to treatment with a ~0.1 M solution of ammonium hexafluorosilicate (99.999%, Sigma-Aldrich, St. Louis, MO, USA) under continuous stirring for 15 min at 80 °C. The powder was then centrifuged and washed multiple times with DI water. The zeolite cake was then dried under N_2_ flow in the furnace at ~100 °C and subsequently treated at 550 °C for 3 h in flowing dry air. This produced the H-MFI sample titled “H-MFI” for our experiments.

To produce the steamed H-MFI sample (containing extra-framework Al species), H-MFI was subjected to steam treatment at 420 °C in a flow-through quartz reactor with air flowing through a water saturator (25 °C) for 30 min. The sample was cooled in flowing wet air to 200 °C and then held at 200 °C in flowing dry air for 1 h before cooling. This sample is called “steamed H-MFI”.

Other H-zeolites were steamed using a similar method, such as H-SSZ-13 and H-BEA (their infrared spectra are shown in Figure S2).

Rhombus-platelet γ-alumina used was synthesized from aluminum isopropoxide via a hydrolysis method [12]. More specifically, approximately 10 g of aluminum isopropoxide was added to ~50 mL of water with vigorous stirring at 80 °C for 1 h. The mixture was transferred to the 125 mL Teflon liner of a Parr reactor and placed into an oven and kept at 200 °C for 24 h. After cooling to room temperature, the powder was collected by filtration, washed with distilled water, and dried at 100 °C. The as-synthesized boehmite powder was then calcined at 800 °C for 2 h to convert it to rhombus-platelet γ-alumina with a surface area of approximately 70 m^2^/g.

Commercial SBA-200 γ-alumina with a surface area of ~200 m^2^/g was used without additional pretreatment.

Rod-like γ-alumina (with surface area ~70 m^2^/g) was synthesized as according to the previous method at pH ~4 [12].

The in situ static transmission IR experiments were conducted in a home-built cell housed in the sample compartment of a Bruker Vertex 80 spectrometer (Billerica, MA, USA) equipped with an MCT detector and operated at a 4 cm^−1^ resolution. The powder sample was pressed onto a tungsten mesh which, in turn, was mounted onto a copper heating assembly attached to a ceramic feedthrough. The sample could be resistively heated, and the sample temperature was monitored by a thermocouple spot welded onto the top center of the W grid. The cold finger on the glass bulb containing CO (99.995%) was cooled with liquid nitrogen, which was the same treatment that was applied to dinitrogen (loaded in the glass bulb from a nitrogen glove box). Research-grade methane (purity 99.995%) was used. Octane (anhydrous, 99.99% Sigma-Aldrich) was purified with multiple freeze-pump-thaw cycles and stored in a glass bulb. D_2_ (purchased from Cambridge Isotopes) was contained in a glass bulb and purified with a liquid nitrogen trap to remove moisture traces. Research-grade H_2_ was used (99.995% purity).

Microscopy analysis was performed with a FEI Titan 80–300 microscope (FEI Company, Hillsboro, OR, USA) operated at 300 kV. The instrument was equipped with a CEOS GmbH double-hexapole aberration corrector (CEOS GmbH, Heidelburg, Germany) for the probe-forming lens, which allows for imaging with 0.1 nm resolution in scanning transmission electron microscopy mode (STEM). HAADF-STEM images were acquired with a high angle annular dark field (HAADF) detector with inner collection angle set to 52 mrad.

^27^Al MAS NMR measurements were performed at 20 °C on a Bruker 850 MHz NMR spectrometer (Bruker, Charleston, SC, USA), operating with a magnetic field of 19.975 T. The corresponding ^27^Al Larmor frequency is 221.4125 MHz. A single pulse sequence comprised of a π/9 pulse width of 0.3 µs, a recycle delay of 2 s, and an acquisition time of 30 ms was employed to collect the free induction decays (FID). To enhance the intensity of the observed spectral features over the noise, 4096 repetitions were employed for each FID. Each collected FID was subsequently Fourier transformed to the frequency domain where both zero and first order phase corrections were applied. The broad spectrometer background signal was collected with a sample containing no Al species under the same conditions in the same MAS NMR rotor and subsequently subtracted from each Al spectra. The data were simulated for best fit and the intensities of each coordination environment from the simulations were taken together to provide the fractional abundance. The total intensity was normalized to the carefully measured mass of each sample used for the NMR experiment, which was typically ~15 mg. All NMR data were acquired at a sample spinning rate of 18.7 kHz (±5 Hz) and externally referenced to 1.0 M aqueous Al(NO_3_)_3_ (0 ppm). The samples were packed inside 3.2 mm pencil-type NMR rotors (Bruker, Charleston, SC, USA). The rotors were subsequently sealed and placed in vials until transported to the NMR probe.

Catalytic experiments were performed in a quartz flow-through reactor. A total of 75 mg of catalyst powder was loaded in the reactor and thermally pre-treated in the flow of dry air at 500 °C, and then purged with dry helium at this temperature for 30 min. Reactions at 390 °C were also performed. For these, the temperature was quickly increased to the desired temperature (390 °C) and catalytic activity was tested at this temperature without pre-treatment. A UHP 2% Propane in He gas mixture was used for the reaction. The WHSV was maintained at ~20 h^−1^ and the products were quantified by gas chromatography equipped with an FID detector. Propane conversions were <2%. The reaction was performed at temperatures between 500 °C and 390 °C.

## Figures and Tables

**Table 1:** Propane cracking and dehydrogenation activity over H-MFI, steamed H-MFI, and γ-Al_2_O_3_. WHSV ~20 h^−1^. Note that cracking produces methane and ethylene, whereas dehydrogenation produces propylene.

Sample	Cracking Activity (Methane Plus Ethylene) × 10^7^ moles/(g × s); 500 °C	Dehydrogenation Activity (Propylene) 10^7^ moles/(g × s); 500 °C
H-MFI	1.4	1.1
H-MFI steamed (dehydroxylated)	9.3	4.5
Rhombus-platelet gamma alumina	0.1	0.8

## Data Availability

All data is available in the main text or the Supplementary Materials.

## Supplementary Materials

The following are available online at https://www.mdpi.com/article/10.3390/molecules27072352/s1, Figure S1: typical HRTEM images of high surface area (~200 m^2^/g) SBA-200 γ-alumina sample. It consists of abundant platelets can be easily seen (image 1) as well less common less well-defined nanocrystal shapes (image 2). Image 3 shows nanocrystal of SBA-200 with macroscopically defined (110) surface: image 4 shows magnified nanocrystal showing that (110) facet is reconstructed into nano (111) and (100) ridges, completely analogous to all gamma-alumina surfaces, Figure S2: (A). Schematic of rhombus-platelet alumina crystals showing reconstruction of (110) surface into (100 and (111) nano-segments. (B–C). Atomically resolved HRTEM image (110) surfaces of rhombus-platelet γ-alumina. The (110) surface is reconstructed into (111) and (100) facets. This is typical for all gamma-alumina samples. (D). HRTEM observation of an irrational surface of γ-alumina showing that it also contains (100) and (111) nano-segments. (e) HRTEM image of (111) surface facet. Reprinted with permission from Angew. Chem. Int. Ed. 2021, 60, 17522–17530. Copyright Wiley-VCH GmbH 2021, Figure S3: FTIR of the OH region of steamed H-SSZ-13, H-BEA and H-MFI samples showing similar EFAL features for all of them, Figure S4: FTIR of the OH region of SBA-200 and rod-like gamma-alumina samples during thermal treatments. Spectra were recorded at room temperature after specified thermal treatments, Figure S5: Solid-state 27Al NMR of fresh and steamed MFI samples. The band ~33 ppm corresponds to penta-coordinated Al, Figure S6: Solid-state ^27^Al NMR fresh and dehydroxylated rhombus-platelet gamma-alumina sample (SA ~70 m^2^/g). Only tetra, penta and octa-Al sites are present. Upon dehydroxylation, surface octahedral O_5_Al(VI)-OH sites dehydroxylate [12] and form penta-sites. Their small abundance is due to the fact that majority of Al remains in the bulk (tetra and octa-Al sites), and only a smaller fraction of Al sites resides on the surface. As the Alumina nano-particle size decreases, more and more Al surface atoms (in terms of relative surface/bulk ratio) get exposed as evidenced from Figure S5 and discussion in the main text. Reprinted with permission from Angew. Chem. Int. Ed. 2021, 60, 17522–17530. Copyright Wiley-VCH GmbH 2021, Figure S7: FTIR spectra showing the C-H stretching region of gamma-alumina that was not treated at high temperature (the sample was heated at 300 °C under vacuum for 15 min, this treatment is not enough to dehydroxylate O5Al(VI)-OH sites) before and after methane pulse at 200 °C, Figure S8: FTIR spectra in the Al-H stretching region after H_2_ treatment of gamma-alumina (treated at 500 °C), Figure S9: H_2_+D_2_ reaction monitored with mass-spec in static FTIR system at temperature 100 °C. A mixture of H_2_+D_2_ was pre-mixed in ~1.6–1.7 volume ratio and introduced into the infra-red cell with rhombus-platelet gamma-alumina sample (dehydroxylated at 500 °C). Mass 3 corresponds to product of the reaction HD. Mass 2 corresponds to H_2_. Mass 4 corresponds to D_2_.

## Sample Availability

Samples of the compounds are not available from the authors.
