# Supplementary material for: On the Nature of Extra-Framework Aluminum Species and Improved Catalytic Properties in Steamed Zeolites
Source: Molecules. 2022 Apr 6;27(7):2352. doi: 10.3390/molecules27072352 (PMC9000512; doi:10.3390/molecules27072352)
Supplement: Supplementary file 1 [file molecules-27-02352-s001.zip › molecules-1499127-supplementary.pdf]

Supplementary Materials

On the Nature of Extra-Framework Aluminum Species and Improved Catalytic Properties in Steamed Zeolites

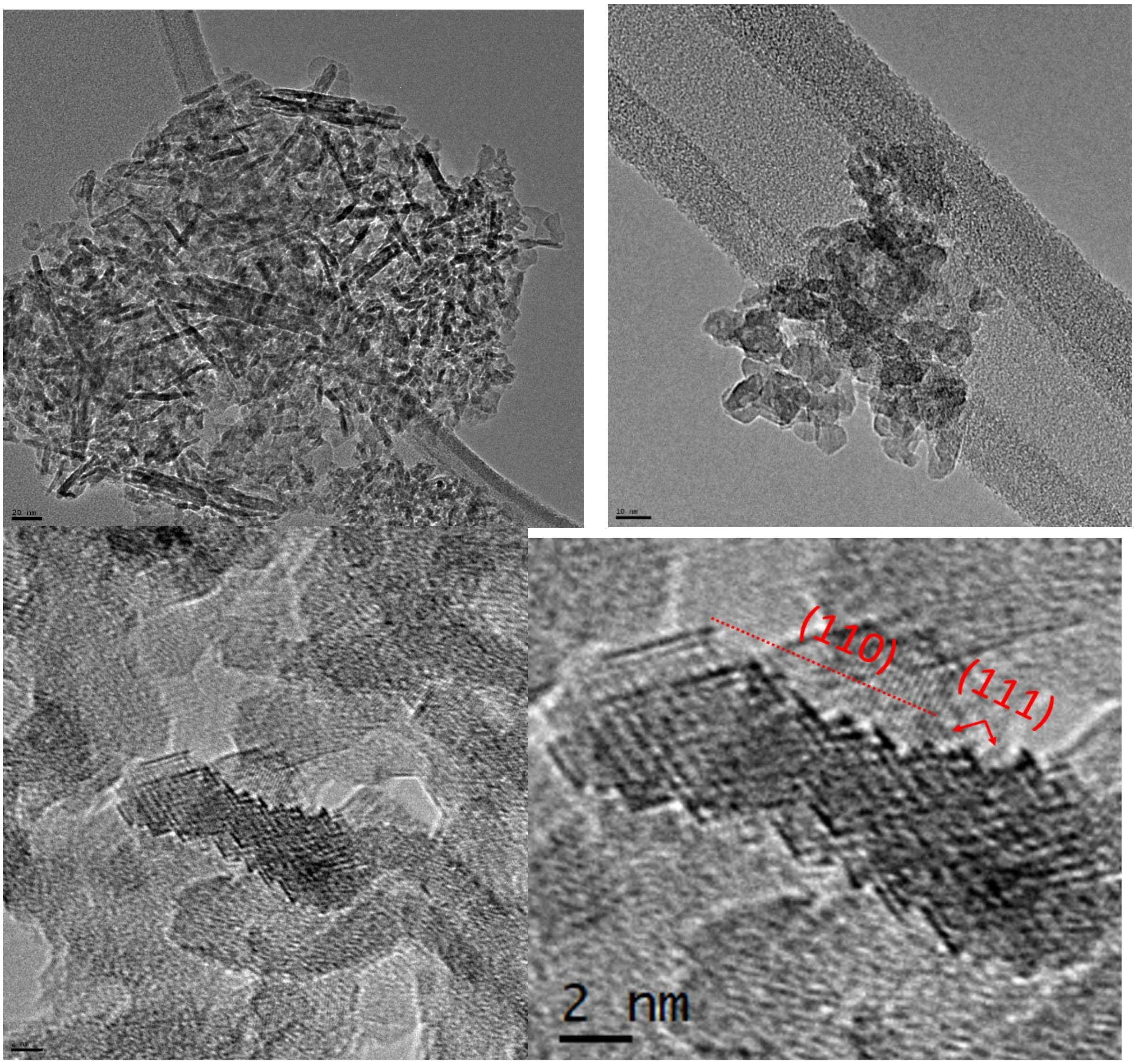

Figure S1. typical HRTEM images of high surface area (~200 m^2/g) SBA-200 γ-alumina sample. It consists of abundant platelets can be easily seen (image 1) as well less common less well-defined nanocrystal shapes (image 2). Image 3 shows nanocrystal of SBA-200 with macroscopically defined (110) surface: image 4 shows magnified nanocrystal showing that (110) facet is reconstructed into nano (111) and (100) ridges, completely analogous to all gamma-alumina surfaces.

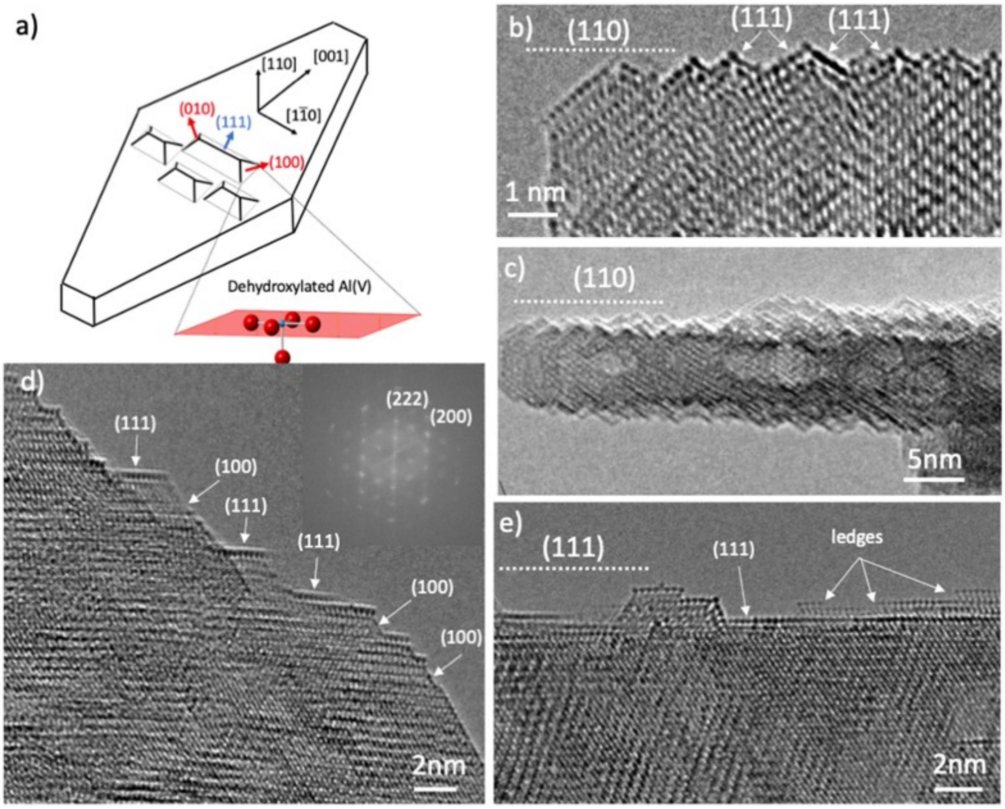

Figure S2. (**a**). Schematic of rhombus-platelet alumina crystals showing reconstruction of (110) surface into (100 and (111) nano-segments. (**b**,**c**). Atomically resolved HRTEM image (110) surfaces of rhombus-platelet γ-alumina. The (110) surface is reconstructed into (111) and (100) facets. This is typical for all gamma-alumina samples. (**d**). HRTEM observation of an irrational surface of γ-alumina showing that it also contains (100) and (111) nano-segments. (**e**) HRTEM image of (111) surface facet. Reprinted with permission from *Angew. Chem. Int. Ed*. 2021, 60, 17522– 17530. Copyright Wiley-VCH GmbH 2021.

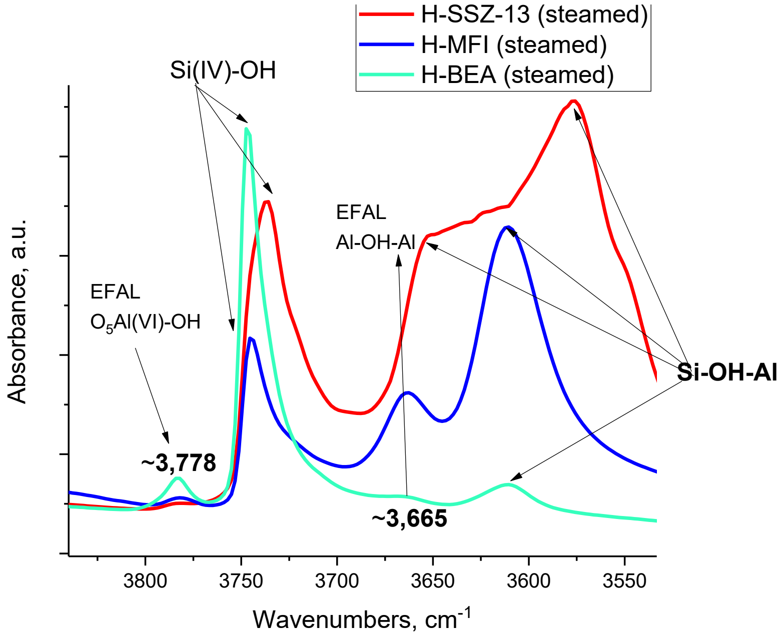

Figure S3. FTIR of the OH region of steamed H-SSZ-13, H-BEA and H-MFI samples showing similar EFAL features for all of them.

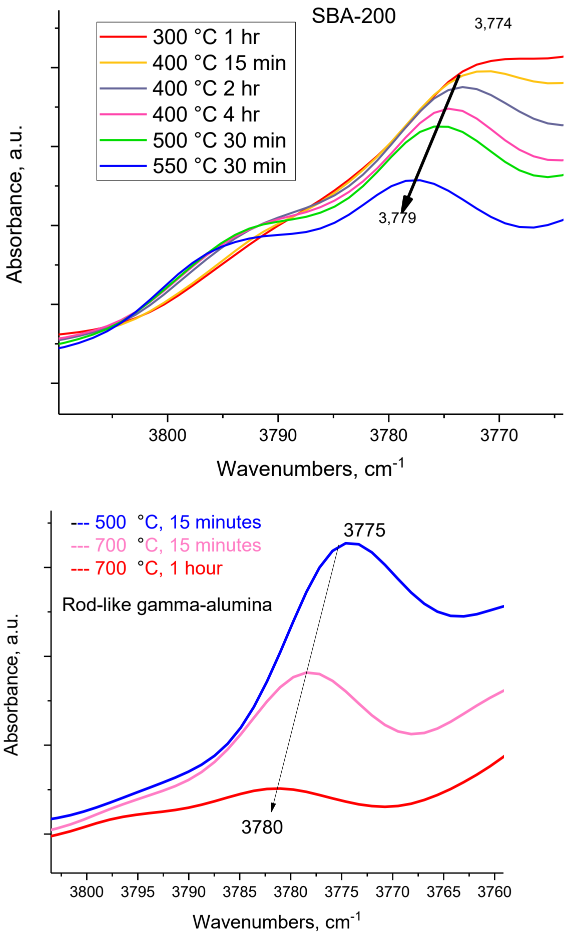

Figure S4. FTIR of the OH region of SBA-200 and rod-like gamma-alumina samples during thermal treatments. Spectra were recorded at room temperature after specified thermal treatments.

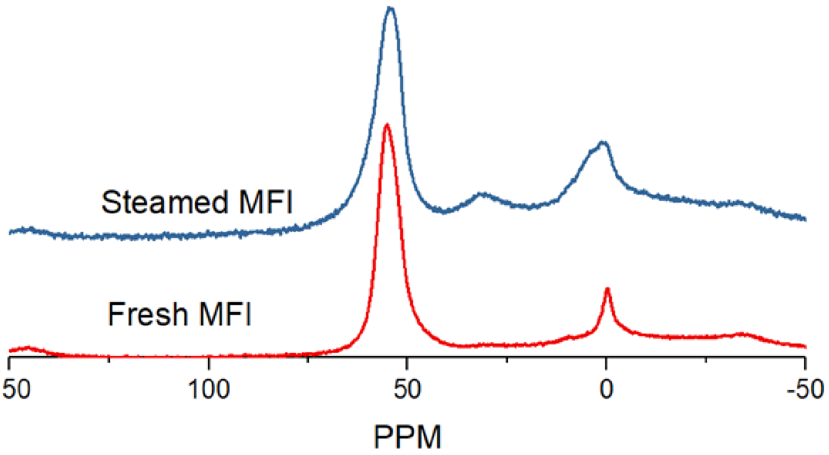

Figure S5. Solid-state ^{27}Al NMR of fresh and steamed MFI samples. The band ~ 33 ppm corresponds to penta-coordinated Al.

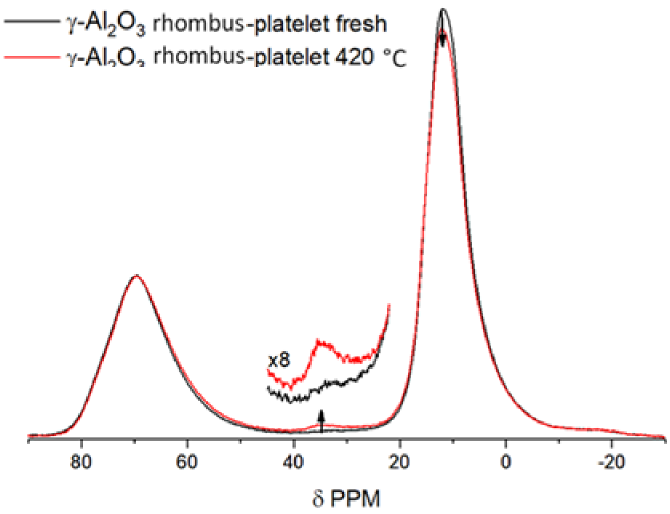

Figure S6. Solid-state ^{27}Al NMR fresh and dehydroxylated rhombus-platelet gamma-alumina sample (SA ~ 70 m^2/g). Only tetra, penta and octa-Al sites are present. Upon dehydroxylation, surface octahedral $O_5Al(VI)$-OH sites dehydroxylate [12] and form penta-sites. Their small abundance is due to the fact that majority of Al remains in the bulk (tetra and octa-Al sites), and only a smaller fraction of Al sites resides on the surface. As the Alumina nano-particle size decreases, more and more Al surface atoms (in terms of relative surface/bulk ratio) get exposed as evidenced from Figure S5 and discussion in the main text. Reprinted with permission from *Angew. Chem. Int. Ed*. 2021, 60, 17522–17530. Copyright Wiley-VCH GmbH 2021.

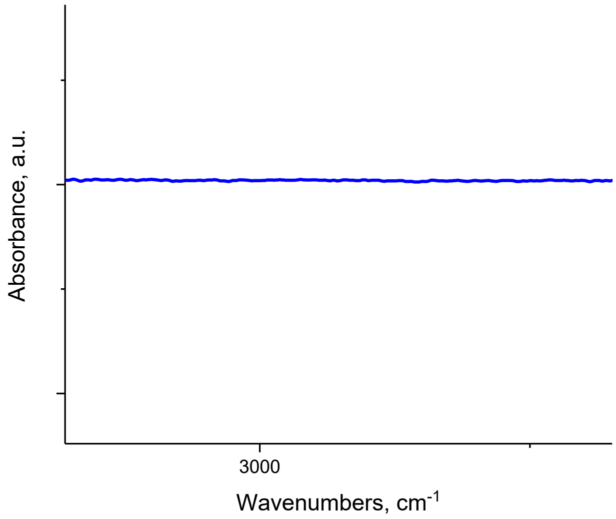

Figure S7. FTIR spectra showing the C-H stretching region of gamma-alumina that was not treated at high temperature (the sample was heated at 300 °C under vacuum for 15 minutes, this treatment is not enough to dehydroxylate $O_5Al(VI)$-OH sites) before and after methane pulse at 200 °C.

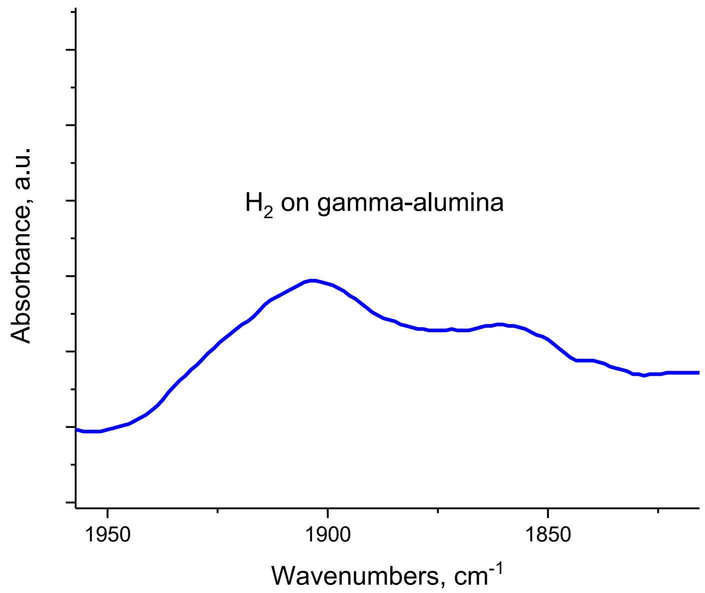

Figure S8. FTIR spectra in the Al-H stretching region after H_2 treatment of gamma-alumina (treated at 500 °C).

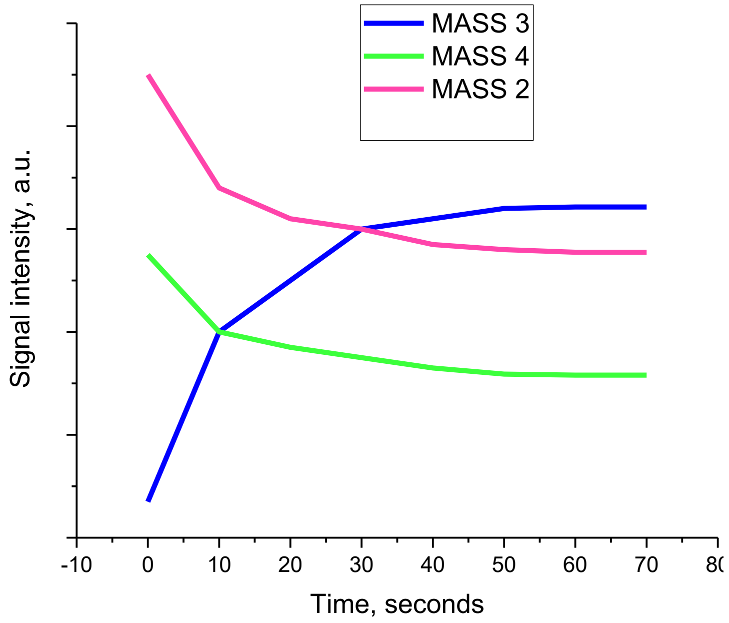

Figure S9. H_2+D_2 reaction monitored with mass-spec in static FTIR system at temperature 100 °C. A mixture of H_2+D_2 was pre-mixed in ~ 1.6-1.7 volume ratio and introduced into the infra-red cell with rhombus-platelet gamma-alumina sample (dehydroxylated at 500 °C). Mass 3 corresponds to product of the reaction HD. Mass 2 corresponds to H_2. Mass 4 corresponds to D_2.
